# Energy–structure coupling mechanism and damage evolution model of red sandstone during soaking–softening process

**DOI:** 10.1371/journal.pone.0341342

**Published:** 2026-01-30

**Authors:** Jingjing Zhang, Ning Liang

**Affiliations:** 1 School of Civil Engineering and Architecture, Guangxi University of Science and Technology, Liuzhou, China; 2 Guangxi Zhuang Autonomous Region Engineering Research Center of Geotechnical Disaster and Ecological Control, Guangxi University of Science and Technology, Liuzhou, China; Guizhou University, CHINA

## Abstract

To reveal how different soaking times affect red sandstone’s softening characteristics, this study analyzed red sandstone’s mineral composition, meso-structure, mechanical properties, and energy evolution laws. A damage constitutive model was established based on mechanical property testing and microstructure determination experiments of rock samples. It considers the initial compaction nonlinear section. The prediction bias in the energy dissipation theory damage model during the compaction stage was corrected based on the correction coefficient. The deterioration of mechanical properties of rock samples is positively correlated with immersion time. The results showed that water soaking caused feldspar, calcite, and other minerals to dissolve. It also reduced clay minerals and made pore development more intense. The mechanical properties of rock samples gradually decrease. This happened as soaking duration increased. When the soaking time reached 150 days, the cumulative deterioration degrees reached 44.25% and 30.78% respectively. The turning point of dissipated energy moved forward. The growth inflection point of the damage variable also advanced. The rock sample damage model fitted well with the experimental curve. It could accurately characterize the softening process. The research results explained the “time–structure–energy–damage” coupling mechanism. This mechanism applies to red sandstone softening under water–rock interactions. The explanation covered both macro and meso perspectives. It provided key theoretical support for red sandstone engineering stability assessment and long-term service safety.

## 1. Introduction

**Fig 1:**
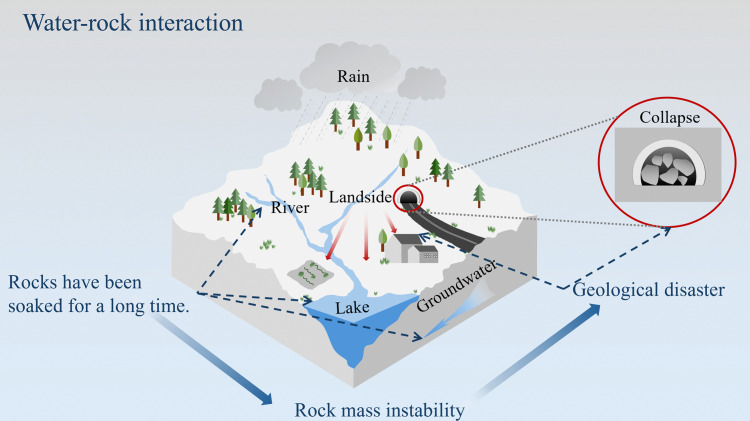
Topographic map.

In the realm of geotechnical research, sandstone usually has a high porosity, and there is often a large amount of pore water in its rock mass. This characteristic makes it extremely prone to phenomena such as argillization, softening, and disintegration [1,2]. The occurrence of these phenomena leads directly to mechanical property deterioration, thereby threatening rock mass stability. When the engineering rock mass is disturbed by dynamic loads such as blasting, earthquakes, and rock bursts, the risk of engineering accidents increases significantly [3–6]. The pore water and dynamic load coupling effect has a profound impact on sandstone stability in projects such as foundations, tunnels, and slopes, as shown in Fig 1. Therefore, in-depth exploration of the degradation characteristics of sandstone under different water exposure durations is crucial for ensuring the stability of sandstone engineering.

Water–rock interaction has long been a core research field in the academic community, with significant advancements achieved in this field. Numerous researchers have conducted research elucidating the variation rules concerning water’s effect on the mechanical properties of rocks [7–10]. At the level of microstructural evolution, relevant studies have revealed how water promotes rock microstructure changes [11–13]. In addition, there have been numerous deliberations regarding the physicochemical reaction mechanisms between water and rocks [14–16]. There have also been numerous advances in research on water–rock interactions in specific rock types. Studies have shown that as soaking time increases, changes in the pore structure of sandstone samples fall into two stages: the clay mineral swelling stage saturation process and the potassium feldspar corrosion stage long-term soaking process [17]. Soaking time alters water quality, weakens sandstone strength, and affects the rock’s failure mode macroscopically [18]. Additional studies have indicated that under different submerged water levels, sandstone strength gradually deteriorates with increasing moisture content and soaking duration [19]. Further specialized research on fractured sandstone has revealed significant changes in its failure characteristics and deterioration mechanism during water-rock interaction: the strength and elastic modulus of saturated sandstone are both lower than those in the natural state, and water-rock reactions additionally alter its failure mode [7,20]. Other research works have indicated that soaking in hydrochemical solutions results in rock mineral dissolution and the formation of substantial internal pores, and these then bring about changes in the macroscopic mechanical properties [21,22]. As for the freeze-thaw effect, as the number of freeze-thaw cycles increases, the roughness of the sandstone fracture surface also increases, and its failure mode changes from transverse fracture to intergranular fracture [23,24]. Another research shows that under the combined action of temperature, water, and stress, as the soaking duration increases, the ratio of sandstone crack initiation stress to peak stress rises and then decreases [25]. A comparative study on the effects of thermal cycling and hydrothermal cycling on the mechanical properties of sandstone shows that there are differences in their effects. Under hydrothermal cycling, the cracks in sandstone are wider, and their impact on mechanical properties is greater [26].

Examining the rock failure mechanism from an energy perspective has become a current research hotspot. Numerous researches have uncovered the tight link between energy and rock failure, holding that accounting for the failure mechanism from this angle is a more rational method [27–31]. For example, a uniaxial test on sandstone soaked in a high–temperature acidic solution reveal that temperature has a greater impact on its energy distribution and failure mode [32]. The three-point bending test conducted on sandstone samples after freeze-thaw cycles showed that Mode I had the highest energy release rate, and its fracture toughness and energy were more sensitive to the influence of cycling compared to other aspects [33,34]. The research on the influence of saturation on the energy evolution of sandstone shows that an increase in saturation will cause changes in energy storage efficiency and capacity [35,36]. Regarding the impact of hydraulic coupling effects on the energy evolution characteristics of sandstone, researchers have proposed a damage constitutive model based on statistical damage theory, which can better describe the mechanical behavior of sandstone under water rock coupling effects [37]. However, despite the rich existing research, there are still obvious knowledge gaps. On the one hand, although many studies have involved various changes in sandstone under water–rock interactions, most of them have not systematically and independently focused on the impact of water on its energy evolution law. The significant water softening characteristics of sandstone are prone to cause complex energy conversion, and existing studies lack in–depth and targeted analysis of this aspect. Meanwhile, regarding a thorough examination of the macroscopic mechanical property degradation of sandstone when exposed to water, meso-structural variations, energy evolution rules, and establishment of a corresponding damage constitutive model, relevant studies are still quite limited and have not fully uncovered sandstone’s deterioration mechanism under water–rock interactions.

Numerous researchers have systematically revealed the damage and deterioration process of rocks from multiple perspectives including elastic modulus, acoustic emission characteristic parameters and energy dissipation. Among these approaches, damage constitutive models based on energy dissipation have been widely applied to describing rock damage processes [38–40]. Specifically, researchers established corresponding damage constitutive models based on the energy evolution laws of gypsum rock with different soaking durations during uniaxial compression [41,42]; for yellow sandstone after wet-dry cycles, researchers constructed a creep damage constitutive model using parameters such as elastic modulus, energy dissipation and plastic strain energy obtained from creep tests [43].

In view of the above research gaps, this study conducted the following research. Firstly, we quantitatively characterized the mineral composition of red sandstone, and comprehensively analyzed the mechanical property deterioration laws, damage evolution, and meso-structural changes in sandstone under different soaking durations. Secondly, based on the energy evolution law of red sandstone during immersion and uniaxial compression, a damage constitutive model considering nonlinear initial compaction section was constructed, and the good fitting degree between the model and the actual results was verified. Finally, we discussed the sandstone deterioration mechanism under water–rock interactions from both the macroscopic and mesoscopic perspectives, aiming to provide a more valuable reference for enriching the theory in the relevant theory regard and ensuring the stability of sandstone engineering projects.

## 2. Materials and methods

### 2.1. Rock sample preparation

**Fig 2:**
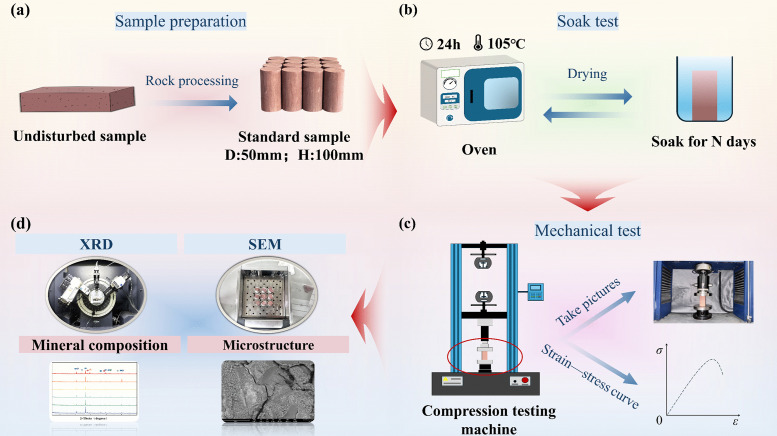
Experimental steps.

The red sandstone chosen in this study was obtained from the southwest region of China. This region features relatively abundant rainfall and widespread rivers and lakes, so its rocks are thus easily impacted by long-term soaking. To guarantee property uniformity, all specimens were harvested from rocks in the same area, and the surface joint characteristics were not prominent. The mineral composition of the specimens was determined via X-ray diffraction analysis. The results indicated that the main mineral components were quartz (41.3%), albite (20.1%), calcite (5.2%), anorthite (9.1%), potassium feldspar (11.1%), and clay minerals (13.2%). This composition indicated that coarse–grained minerals (quartz and feldspar) formed the main framework of the rock, while clay minerals were attached to the surface of these, playing a cementing and filling role. In line with the criteria of the International Society for Rock Mechanics (ISRM) [44], the rock mass was drilled, cored, sliced, and polished to make standard cylindrical specimens. The sample size was Φ 50 mm × H 100 mm, as shown in Fig 2a.

### 2.2. Test methods

We grouped rock samples for softening tests. Samples were divided into 6 groups according to strict standards, with 3 samples in each group to facilitate systematic comparative analysis. Each group was placed in a temperature–controlled oven at 105°C and dried continuously for 12 hours to ensure that each rock sample reached a relatively consistent initial dry state; in this way, we could eliminate the possible impact of differences in the natural moisture content on the test results [7]. This research focuses primarily on the irreversible damage characteristics of red sandstone after water immersion. For this purpose, after soaking, the specimens were again oven-dried at a constant temperature of 105°C for 24 hours to eliminate the impact of moisture content differences on the mechanical properties of red sandstone, with the specific process illustrated in Fig 2b.

For the mechanical performance test of each group of samples, a computer-controlled electronic universal testing machine was adopted. The loading rate was precisely set to 0.05 mm/min, ensuring stability and uniformity during the testing process, thereby obtaining reliable and accurate data. To ensure test repeatability, 3 independent uniaxial compression failure tests were conducted based on the above test parameters for each group of samples immersed for the same duration, as shown in Fig 2c.

After the mechanical performance test, the fractured samples were further processed and inspected to determine structural damage. Firstly, sample fragments were meticulously pulverized into a uniform powder for X-ray diffraction (XRD) tests to examine the subtle changes in their internal mineral components. In addition, sample surface failure images were captured to record the crack propagation state after the samples reached peak stress and watch crack development and morphological characteristics under different soaking durations. Furthermore, by means of scanning electron microscopy (SEM) observation equipment, the changes in the internal structure of the sample fracture surfaces, like pore development, crack expansion and the connection state variation among mineral particles, were closely observed. By doing so, we sought to uncover the damage development mechanism of red sandstone during the water softening process at the microscopic level, as displayed in Fig 2d.

## 3. Test results and analysis

### 3.1. Change in mineral composition in rock sample softening

**Fig 3:**
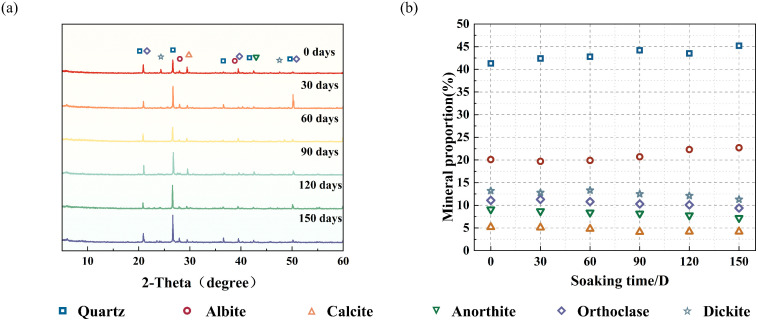
X-ray diffraction results of pattern of red sandstone for different immersion times. **(a)** X–ray diffraction pattern. **(b)** Mineral composition content.


The XRD outcomes of the samples under different soaking durations are presented in Fig 3a. In comparison with the samples soaked for 0 days, the peak intensities and areas of various mineral components underwent minor changes. The proportions of quartz and albite rose during soaking, while the diffraction peak intensity of clay minerals gradually declined with increased soaking time. The content proportions also decreased accordingly, which matches prior research findings [45]. After soaking for 150 days, the content change in a single mineral among the six mineral components did not exceed 5%, as shown in Fig 3b. Distilled water causes some mineral components, such as potassium feldspar, anorthite and calcite, to dissolve. The chemical reaction equations are shown below:

$$KAlSi_{3}O_{8}+4H^{+}=Al+3SiO_{2}+2H_{2}O+K^{+}\mathrm{\backslash ]}$$ (1)

$$CaAl_{2}Si_{2}O_{8}+8H^{+}=2Al^{3+}+2H_{4}SiO_{2}+Ca^{2+}\mathrm{\backslash ]}$$ (2)

$$CaCO_{3}+2H^{+}=Ca^{2+}+H_{2}O+CO_{2}↑\mathrm{\backslash ]}$$ (3)

The above reactions indicate that hydrolysis, ionization and dissolution reactions occur among the minerals inside the samples, meaning that the mineral components dissolve in water and attach to the pore walls to form new precipitates, thus changing the original pore structure. Meanwhile, water–rock interactions form a continuous process. As the soaking time increases, the content of dissolved minerals in the pores rises, leading to higher alkalinity, which in turn speeds up the minerals’ dissolution process and further worsens the deterioration and damage to the internal structure of the samples [17].

### 3.2. Mechanical characteristics of rock sample failure process

**Fig 4:**
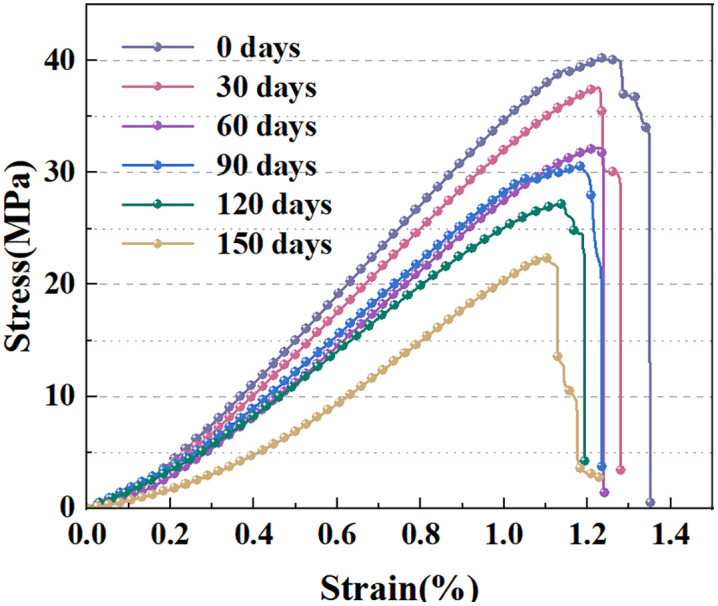
Uniaxial mechanical test curves of red sandstone for different immersion times.

Fig 4 shows the stress-strain curves of samples with six different soaking periods in uniaxial compression testing. At different stages, the stress-strain evolution patterns of the samples exhibit similar trends, including initial compaction, linear elasticity, crack development, and failure stages [13,46].

The stress–strain curves of samples with various soaking periods all exhibit notable nonlinear concave characteristics at the initial loading stage. This stems from the rapid closure of the samples’ original pores under external loading [13]. As the soaking time increases, the plastic deformation of the rock sample gradually increases, resulting in a significant extension of the initial compaction stage. When the load is further increased, the curve shape gradually tends to be linear or approximately linear, and its tangent slope drops with increasing soaking duration, suggesting that the elastic modulus of the specimen is inversely proportional to the soaking duration. At the peak strength stage, the samples soaked for 0 days exhibit the highest compressive strength, and the uniaxial compressive strength (UCS) presents a declining tendency with prolonged soaking. The decrease in compressive strength is especially notable in the samples soaked for 60 days. It should be noted that there is a significant correlation between the decreasing trend of tangent slope in the linear elastic stage and the expanding range of initial compaction and yield strengthening stages.

### 3.3. Deterioration characteristics in rock sample softening

To characterize the effect of varying soaking durations on the mechanical behaviors of samples, a method for quantifying the deterioration degree was proposed based on the deterioration mechanism of red sandstone mechanical parameters. The cumulative degree of deterioration under a certain soaking duration is defined as the total deterioration degree. The deterioration percentage of a single mechanical parameter is defined as follows, as shown in Equations (4) and (5).


$$S_{N}=\frac{T_{0}-T_{N}}{T_{0}}\times 100\%$$ (4)

$$\Delta S_{N}=S_{N}-S_{N-1}$$ (5)

**Fig 5:**
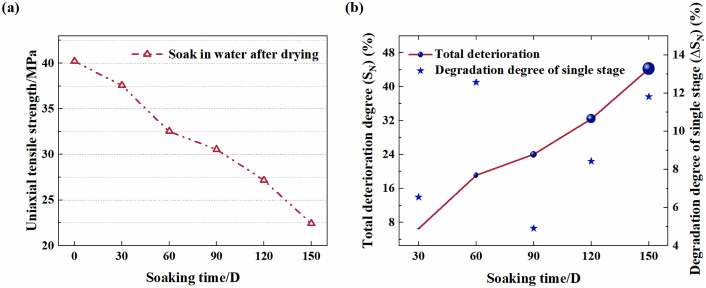
The degradation characteristic curve of compressive strength of red sandstone. **(a)** Deterioration curve. **(b)** Single order and cumulative degradation degree.

In Equations (4) and (5), *T*_0_ represents the mechanical parameter of the sample soaked for 0 days, and *T*_*N*_ represents that for N days. The deterioration characteristics of the peak stress and elastic modulus of samples under different soaking durations are shown in Figs 4 and 5, respectively.

It can be seen from Fig 5a that the UCS of samples shows a stepwise decreasing trend with increasing soaking duration. Among these, when soaked for 30 days, the cumulative deterioration degree of the UCS of the sample is $S_{30}=6.54\%$. As the soaking time increase, the deterioration degree gradually increases and reaches $S_{150}=44.25\%$ when soaked for 150 days, as shown in Fig 5b. The UCS deterioration percentages (Δ*S*_*N*_) corresponding to the samples with different soaking durations from 30 days to 150 days are 6.54%, 12.57%, 4.91%, 8.43% and 11.81%. In turn, this indicates that long–term soaking has a significant weakening effect on the UCS of sample.

**Fig 6:**
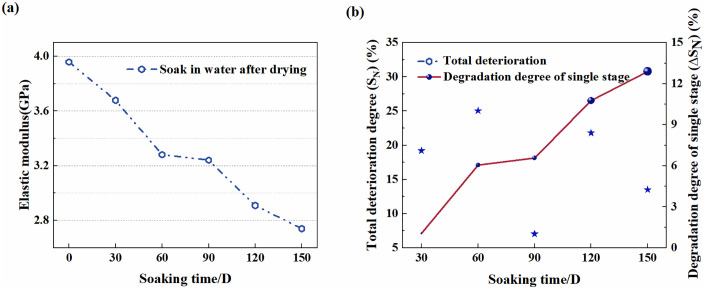
Characteristic curve of degradation of elastic modulus of red sandstone. **(a)** Deterioration curve. **(b)** Single order and cumulative degradation degree.

Fig 6a shows that the elastic modulus (*E*) of samples shows a deteriorating and decreasing trend similar to that of the UCS with increasing soaking duration. It can be seen from Fig 6b that the cumulative deterioration degree *S*_*N*_ increases cumulatively over time and reaches 30.78% after soaking for 150 days. It is worth noting that $S_{60}=17.12\%$ and $S_{90}=18.13\%$, with a difference of only 1.22% between them. The elastic modulus deterioration percentages (Δ*S*_*N*_) corresponding to the samples with different soaking durations from 30 days to 150 days are 6.54%, 12.57%, 4.91%, 8.43% and 11.81%. Notably, the soaking-induced weakening is slightly more pronounced for the UCS than for the *E*, with both deterioration trends exhibiting a distinct time dependence characterized by a positive correlation between the degree of deterioration and soaking duration.

## 4. Energy analysis and evolution model of rock sample damage

### 4.1. Strain energy calculation method

**Fig 7:**
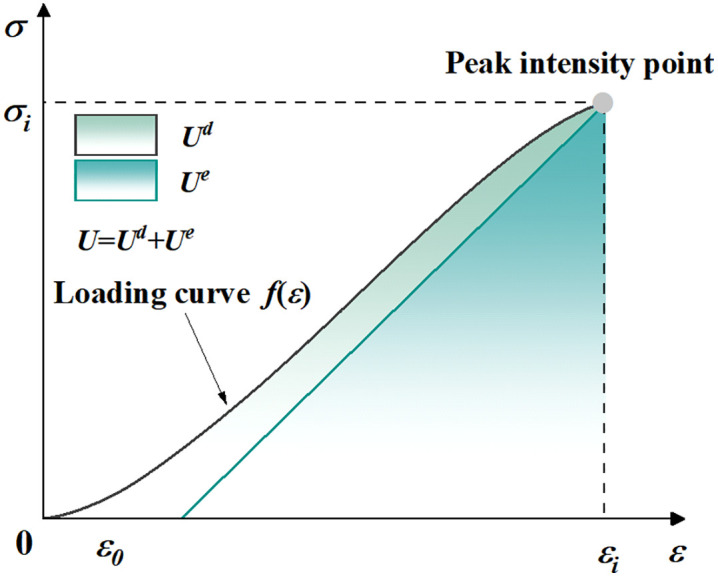
Evolution characteristics of red sandstone strain energy.


During the compression failure process, the energy of the rock sample undergoes several different stages. The test apparatus achieves energy input by applying axial loading; the rock continuously stores energy in the form of elastic strain energy and dynamically completes energy dissipation and release during loading. As shown in Fig 7, during uniaxial compression, the area enclosed by the stress-strain curve and coordinate axis represents the total energy accumulated and released during that stage. This is usually calculated through the micro element integration method [32,47], whose formula is as follows:

$$U=\int_{0}^{\varepsilon }\frac{\sigma_{i}+\sigma_{i+1}}{\text{2}}d\varepsilon =U^{d}+U^{e}$$ (6)

In Equation (6), *U* is the total absorbed energy, $\sigma_{i}$ and $\sigma_{i+1}$ are the stresses of the trapezoidal micro–unit, ε is the elastic strain of peak strength, $U^{e}$ is the stored elastic strain energy, and *U*^*d*^ is the dissipated energy.

The elastic energy before the peak is shown in Equation (7):


$$U^{e}=\frac{\varepsilon_{e}}{\text{2}}\sigma_{c}$$ (7)

In Equation (7), $\varepsilon_{e}$ is the elastic strain of peak strength and $\sigma_{c}$ is the sample’s peak strength. *U*^*d*^ can be calculated according to Equation (8):


$$U^{d}=U-U^{e}=\int_{0}^{\varepsilon }\frac{\sigma_{i}+\sigma_{i+1}}{\text{2}}d\varepsilon -\frac{\varepsilon_{e}}{\text{2}}\sigma_{c}$$ (8)

### 4.2. Energy evolution characteristics of rock softening

**Fig 8:**
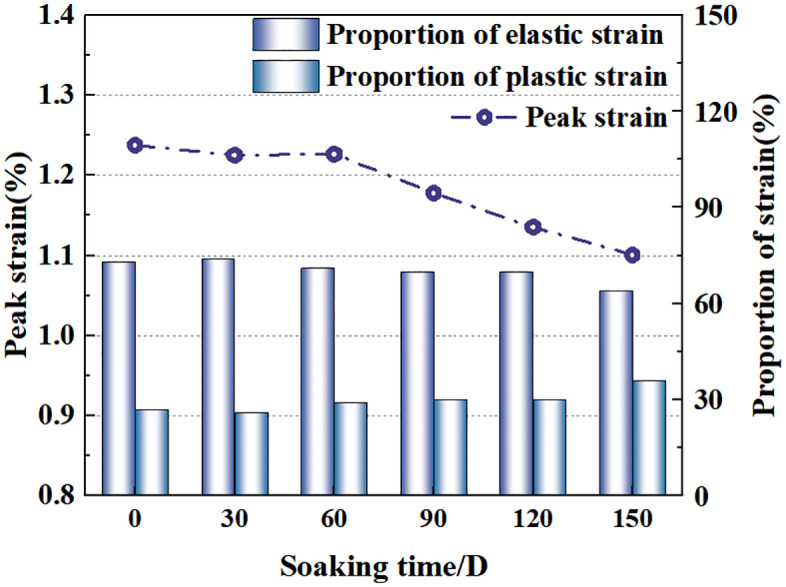
Distribution of plastic strain and elastic strain ratio before peak of rock sample.

Based on the uniaxial compression test data from samples with different soaking durations, the pre–peak strain evolution laws were revealed, as shown in Fig 8. The proportion of elastic strain shows a generally decreasing trend with increasing soaking duration, with specific values being 73% (0 days), 74% (30 days), 67% (60 days), 66% (90 days), 70% (120 days), and 60% (150 days). Correspondingly, the proportion of plastic strain increased to 36% at 150 days. It can be seen from Fig 8 that the peak strain of the 0 days sample is slightly higher than those of the 30 days and 60 days samples. However, it generally shows a significant decreasing trend with increasing soaking duration. After soaking for 120 days, the softening effect of water promoted increased plastic strain.

**Fig 9:**
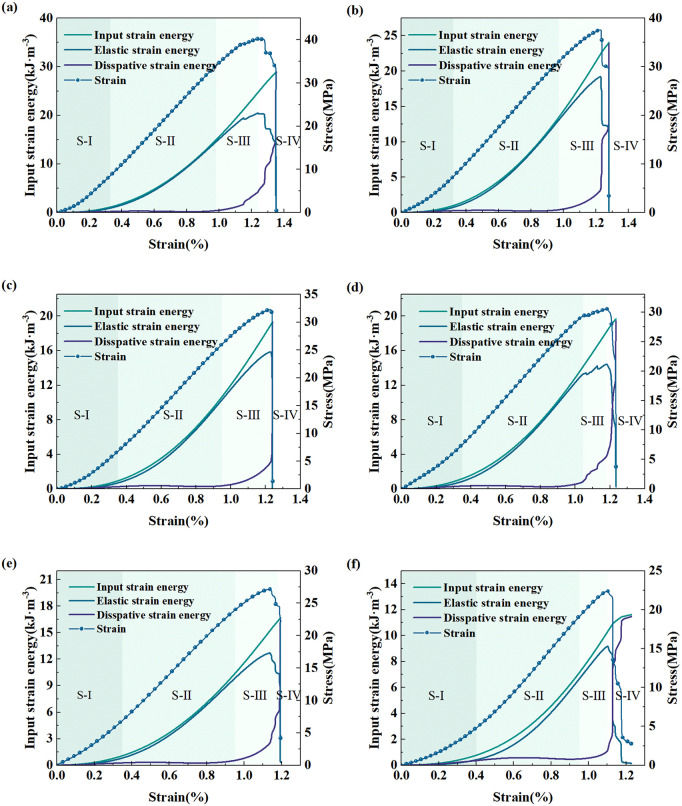
Energy evolution characteristics of rock samples. **(a)** Soaking for 0 d. **(b)** Soaking for 30 d. **(c)** Soaking for 60 d. **(d)** Soaking for 90 d. **(e)** Soaking for 120 d. **(f)** Soaking for 150 d.

As shown in Fig 9, the degradation mechanism and energy evolution characteristics of red sandstone under different soaking times are revealed. Deformation and failure of samples in the uniaxial compression procedure can be classified into four stages: micro–crack closure (S–I), elastic stage (S–II), macroscopic crack propagation stage (S–III) and post–peak stage (S–IV).

(1) Initial compaction stage (S–I): Some of the total energy input via applying axial loads is reserved in the form of elastic energy; however, a portion of this is used up and released because of the closure of the original cracks inside the sample [48], and the dissipated energy curve therefore presents a slowly increasing trend.
(2) Elastic deformation stage (S–II): As the axial stress increases, the cumulative total energy rate rises remarkably, its evolution curve grows nearly synchronously with the elastic strain energy curve. The absorbed energy is stored as elastic strain energy, only a small quantity of energy is dissipated. This is because, in this stage, nearly no new micro–cracks are formed or the original cracks extend, and part of the energy is consumed and released due to friction among particles [48].
(3) Crack initiation and propagation stage (S–III): The elastic energy evolution curve no longer grows linearly or continuously, and its curve slope starts to decline. The dissipated energy evolution curve slope and proportion start to gradually rise. This is because new micro-cracks gradually form, extend, intersect and connect, and the friction among cracks increases, consuming more energy. Since the sample is still continuously taking in external work, the total energy evolution curve is still rising, and the elastic energy and total energy evolution curves no longer keep a synchronous developing state.
(4) Post–peak failure stage (S–IV): After the sample attains its peak strength, the elastic strain energy curve falls rapidly, and the dissipated energy curve rises rapidly. This is because the rock suddenly experiences brittle failure when the external load surpasses the peak stress, and the elastic strain energy is released quickly and massively. A large number of internal micro–cracks link and propagate to form macroscopic cracks, leading to rock sample breakage and invalidation [49]. Specifically, when the soaking duration reaches 150 days, the variation trend of dissipated energy no longer shows a steep rise but shifts to a stepwise increase.

**Fig 10:**
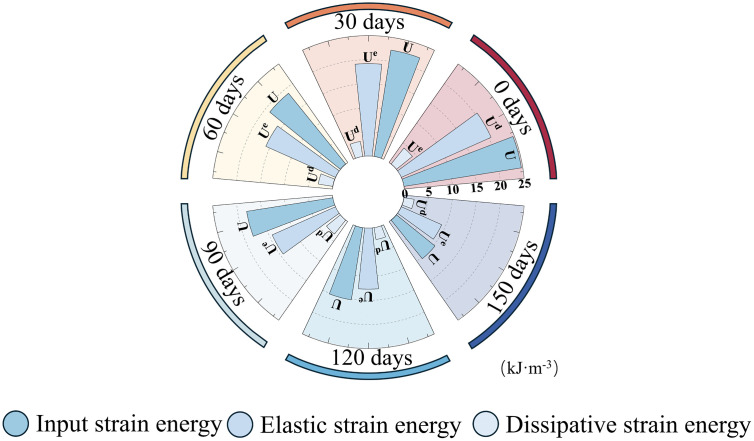
Energy evolution parameter values before peak of rock samples.

Under uniaxial compression, the energy evolution law of red sandstone has a notable correlation with soaking duration. The total energy needed by the sample before reaching peak strength slowly declines over time, and the turning point of dissipated energy shifts earlier, as presented in Fig 10. This suggests that the softening effect of water impairs the energy storage capacity of red sandstone. This phenomenon is highly associated with the meso-structural weakening of the sample, such as cement dissolution and pore connection [11,18]. As shown in Table 1, the proportion of elastic strain energy is still the main part of the total strain energy (>79%), yet its capacity to gather elastic strain energy slowly reduces as soaking duration increases. The elastic strain energy of the 150-day sample drops by 55.10% in comparison to that of the 0-day sample.

**Table 1:** Energy evolution parameter values before peak of rock samples.

Soaking time	*U*(kJ·m^–3^)	*U*^*e*^(kJ·m^–3^)	*U*^*d*^(kJ·m^–3^)
**0 d**	24.69	20.42	4.27
**30 d**	22.34	19.20	3.14
**60 d**	18.89	15.84	3.05
**90 d**	18.13	14.40	3.73
**120 d**	15.16	12.68	2.48
**150 d**	10.30	9.17	2.13

### 4.3. Damage evolution equation considering compaction effect

According to the Lemaitre strain equivalence assumption, the damage constitutive model of the rock sample can be expressed as follows [50–52]:


$$\sigma_{L}=\left(1-D\right)E\varepsilon$$ (9)

In Equation (9), *E* is the elastic modulus, *D* is the damage variable, and $\sigma_{L}$ is the model stress value.

To characterize the damage accumulation and performance deterioration process caused by crack development and rock material expansion under loading states, previous studies have defined damage variables from different perspectives [53–55]. Rock damage and fracture are accompanied by irreversible energy dissipation and release. Assuming no heat exchange with the external environment, the energy dissipation process accurately reflects the damage evolution [51]:


$$D=\frac{U^{d}}{U^{dm}}$$ (10)

In Equation (10), *U*^*d*^ is the cumulative dissipated energy value under any given stress state, and $U^{d}\in [0,U^{dm}]$. *U*^*dm*^ is the cumulative dissipated energy value when the rock is finally completely destroyed. When *D* = 0, this indicates that the rock is intact without damage, and when *D* = 1, this indicates that it is damaged and has completely lost its bearing capacity.

Considering that the rock still has a certain bearing capacity after the peak and has not been completely destroyed, the above formula can be modified as follows:


$$D=\left(1-\frac{\sigma_{r}}{\sigma_{p}}\right)\frac{U^{d}}{U^{dm}}$$ (11)

In Equation (11), $\left(1-\sigma_{r}/\sigma_{p}\right)$ is the correction factor, $\sigma_{r}$ is the residual stress, and $\sigma_{p}$ is the peak stress.

The Lemaitre damage constitutive model is obtained:


$$\sigma_{L}=\left(1-\left(1-\frac{\sigma_{r}}{\sigma_{p}}\right)\frac{U^{d}}{U^{dm}}\right)E\varepsilon$$ (12)

The mechanical properties of naturally heterogeneous rock materials are affected by original micro–cracks, as well as internal crack closure appearing in the early stage of loading. Therefore, it is necessary to modify the constitutive equation to better conform to the actual test curve. Currently, the constitutive equation can be modified in two ways. One is to modifying the original constitutive equation by introducing a correction coefficient, and the other is to obtain the modified strain by subtracting the crack closure strain from the total strain [56]. In this study, a new correction coefficient *K* is introduced and defined. The calculation formula is as follows:


$$K=\frac{E_{S}}{E_{SL}}$$ (13)

**Fig 11:**
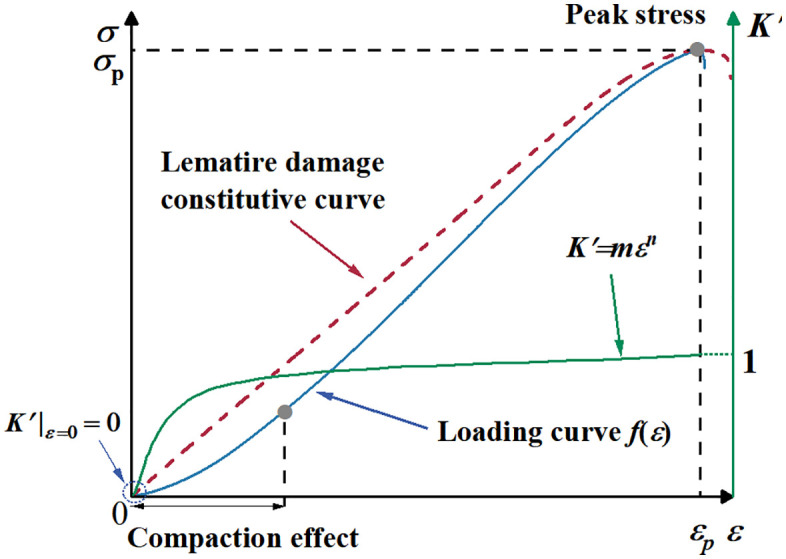
Comparison between the traditional constitutive and actual curves.


The initial value of *K* is assumed to be 0, and the nonlinear evolution between *K* and strain can be quantitatively described by a power function, as shown in Fig 11.

$$K=m\varepsilon^{n}$$ (14)

In Equation (14), *m* and *n* are constants obtained from experiments. Substituting Equation (14) into Equation (12), a new modified constitutive model can be obtained:


$$\sigma_{m}=K\left(1-\left(1-\frac{\sigma_{r}}{\sigma_{p}}\right)\frac{U^{d}}{U^{dm}}\right)E\varepsilon$$ (15)

In Equation (15), $\sigma_{m}$ is the modified stress obtained from the above modified model above.

### 4.4. Damage evolution law of rock sample compression failure

**Fig 12:**
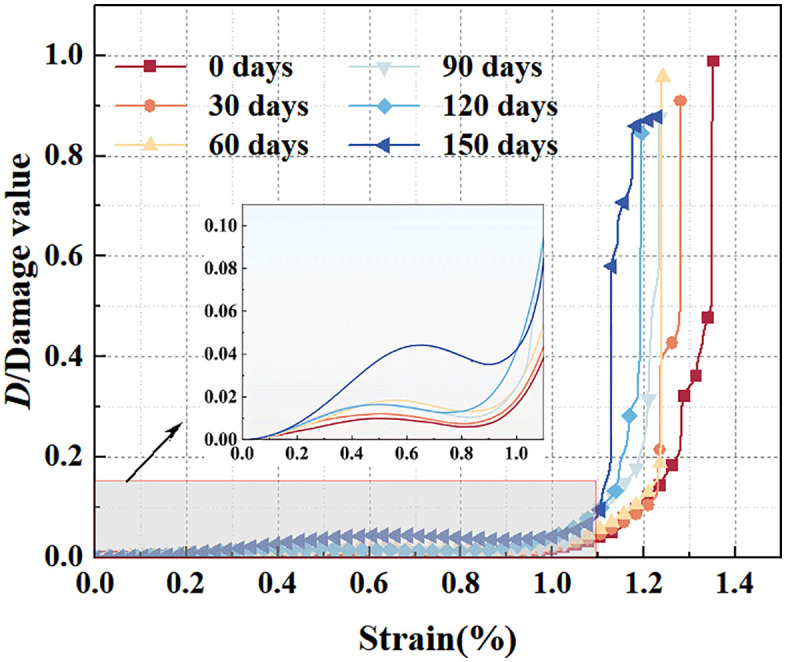
Evolution of damage variable of red sandstone for different immersion times.

Through the cumulative dissipated energy *U*^*d*^ when the sample is completely destroyed and the residual strength $\sigma_{r}$ can be obtained via the mechanical test. The relevant data are shown in detail in Table 2. Damage evolution curves under different soaking durations can be calculated using Equation (8), and their overall trend shows an “L” shape tilting to the left, as shown in Fig 12. It can be found that with increasing soaking time, the key inflection point of the growth of the damage variable gradually moves forward. The growth interval of the damage variable becomes smaller and smaller. This indicates that with water’s softening effect, internal sample damage of samples appears earlier.

**Table 2:** Experimental parameters for the constitutive mode.

Days	*E*(GPa)	*σ*_*p*_(MPa)	*σ*_*r*_(MPa)	*ε*_*p*_(%)	*m*	*n*	*R* ^*2*^
**0**	3.96	40.21	0.47	1.24	0.91	0.32	0.9916
**30**	3.68	37.58	3.42	1.23	0.90	0.31	0.9940
**60**	3.28	32.25	1.38	1.23	0.885	0.40	0.9976
**90**	3.24	30.55	3.75	1.18	0.90	0.22	0.9939
**120**	2.91	27.16	4.17	1.14	0.89	0.20	0.9956
**150**	2.74	22.42	2.75	1.10	0.52	0.77	0.9930

In the initial compaction stage, the original cracks inside the sample are gradually closed under compression, resulting in nonlinear deformation, which can be regarded as a phenomenon of initial damage “recovery”. Initial defects such as original cracks and fissures are compacted and closed under the initial load, generating friction and dissipating a small amount of energy. At this time, the damage variable is close to 0, indicating that the sample undergoes almost no damage in the initial stage. In the elastic deformation stage, the sample undergoes elastic deformation. After the load exceeds the crack closure stress, the original defects are closed without the initiation or propagation of new cracks. Therefore, as the strain increases, the damage variable increases slowly and then remains almost unchanged. In the crack initiation and growth stage, as the load continues to increase but has not yet reached the peak strength, new micro–cracks begin to appear, gradually initiating, expanding and intersecting and connecting inside the sample. Damage then accelerates, and the damage evolution curve enters a nonlinear evolution stage.

**Fig 13:**
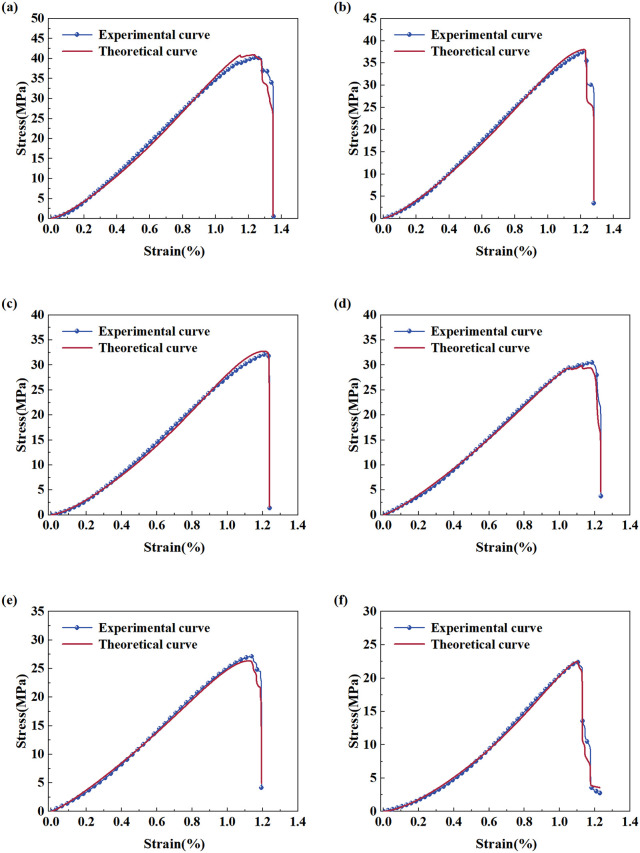
Theoretical and experimental stress–strain curves of red sandstone for different immersion times. **(a)–(f)** showing water immersion times for 0, 30, 60, 90, 120, and 150 days, respectively.

As shown in Fig 13, by substituting *D* and *K* into Equation (15), the theoretical damage constitutive curves of samples can be obtained. Comparison of the theoretical and experimental curves shows that under different soaking durations, the theoretical constitutive curves basically coincide with the experimental ones. This result indicates that the rock damage constitutive model established based on the compaction effect can not only better simulate the stress–strain relationship of the rock under uniaxial compression conditions, but can also better reflect the nonlinear fitting results of the initial compaction section of the samples.

## 5. Discussion

### 5.1. Rock sample softening failure mode

**Fig 14:**
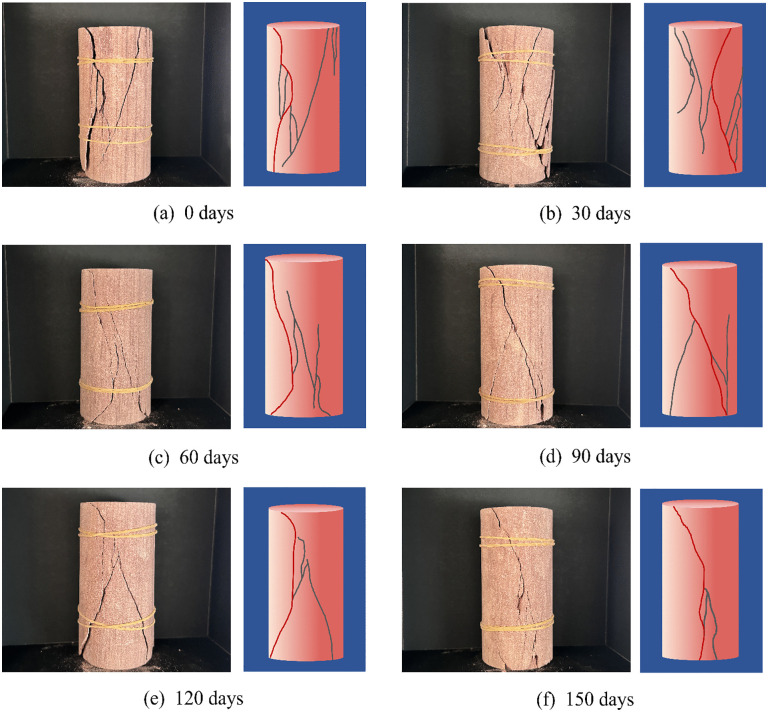
Sample failure characteristics under different water immersion conditions.

Fig 14 shows the compression failure modes of rock samples at different soaking times. When the rock samples soaked for 0 and 30 days were destroyed, there was large block ejection and the degree of fracture was the most severe. The samples split and fractured into two parts, with large fragments, a long ejection distance, and explosive sounds. Shear and splitting cracks also appeared near the main through crack. The surface crack morphologies of the samples soaked for 60, 90 and 120 days presented a “Δ” shape, showing a composite splitting–shear failure mode. Compared with the samples soaked for 0 and 30 days, these samples had fewer secondary splitting cracks near the main crack, a lower fragment ejection force, and a duller sound when failing. For the sample soaked for 150 days, a through splitting crack was generated on its surface, showing typical splitting failure and obvious plastic characteristics, without fragment ejection, and the sound was dull.

It can be seen from Fig 14 that the samples soaked for 0 and 30 days had obvious original defects. During the loading process, lateral tensile stress was easily generated inside the samples; they had a high degree of fragmentation, the debris was small, and they showed brittle failure characteristics. For the samples soaked for 60, 90 and 120 days, the development of pores and cracks was unbalanced, and most of the cracks on the sample surfaces were splitting. As the soaking time increases, the local defects of the rock sample become more and more obvious, mainly manifested as splitting failure. Meanwhile, the interconnection of local cracks induced secondary cracks. After soaking for 150 days, the best weak surface was formed inside the rock sample, which became the fracture surface of the sample.

### 5.2. Analysis of meso-structural changes in rock samples

**Fig 15:**
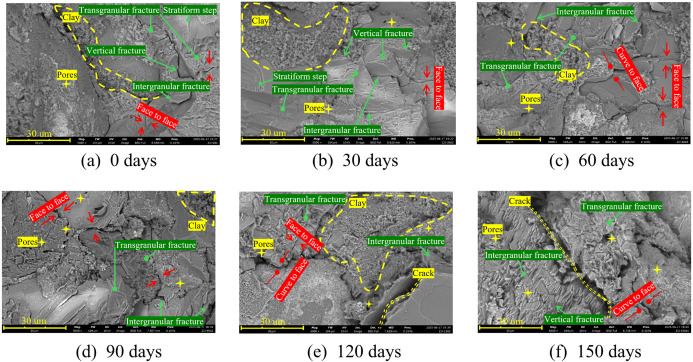
Meso-failure structure of red sandstone with different immersion times (×5000).

In this study, by analyzing the changes in the meso-structure of samples after soaking, as well as the fracture characteristics after uniaxial compression tests, the deterioration mechanism of red sandstone under water–rock interaction was explored. SEM scanning images of samples soaked for different durations are shown in Fig 15. The results show that the 0 days sample had fewer pores, and the cementing materials between aggregate particles present a porous sponge–like structure, resulting in the sample having good water absorption. With increasing soaking time, the internal cementing degree, pores and fracture characteristics have all changed significantly. As soaking time increases, the red sandstone exhibits blurred edges of mineral particles, tiny corrosion pits formed on the surface of framework minerals, slow dissolution of clay minerals, and other phenomena. This result confirms the occurrence of water-rock interaction and is consistent with the XRD test results.

(1) After soaking for 0 and 30 days, the basic framework of the samples was relatively intact, with local fracture phenomena and relatively large broken particles. The contact mode between particles is mainly face–to–face contact, and cementing lines can be seen. The fragmentation degree at 30 days is higher than that at 0 days. The samples show intergranular fracture and trans–granular fracture, mainly trans–granular fracture. In addition, they also show vertical fracture and cleavage step characteristics. With the dissolution effect of water on the mineral components in the samples, pores and micro–cracks begin to develop and initiate.
(2) After soaking for 60 days, the number of sample debris particles increases, the skeleton is broken and partially fragmented, micro–cracks expand significantly, and the meso-failure shows a trans–granular fracture → intergranular fracture mode. Particles are stratified, and the peeling phenomenon is obvious. Pores and cracks continue to develop, which means that the dissolution and erosion of red sandstone by water weakens the cementation between particles, leading to particle detachment. Therefore, the interaction between water and rock accelerates the development of pores inside red sandstone, destroys the microstructure of the sample, and leads to the deterioration of mechanical properties.
(3) After soaking for 150 days, water erosion causes the aggregate particles to peel off, exposing new water–rock interfaces, making the surface rough and promoting pores and crack development. The erosion of the sample’s meso-structure makes the layered structure obvious, the particle arrangement presents a stepped shape, and the contact mode changes to line–to–face contact. Meanwhile, erosion fracture zones appear on a large scale, running through the entire observation area. From a mesoscopic perspective, the interaction between water and rock is mainly reflected in the weakening of cementation effect, changes in the contact mode between particles, promotion of pore and crack development, continuous expansion of water rock contact surface, and exacerbation of the deterioration of sample meso-structure.

### 5.3. Rock sample damage mechanism during softening

In the process of water rock interaction, red sandstone undergoes damage evolution mainly due to physical and chemical effects [17,57,58]. These include increased pore water pressure, chemical reactions, decreased intergranular cohesion and friction, reduced fracture energy, and the dissolution and expansion of mineral components. Specifically, soaking weakens the interparticle bonding strength of red sandstone, promoting the formation of microcracks along weak cementation surfaces. As soaking time increases, the specimen structure becomes increasingly loose, the energy required for failure decreases, and the failure mode transforms from brittleness to ductility. Continuous accumulation of microstructural damage ultimately leads to the progressive degradation of the specimen’s macroscopic mechanical properties.

**Fig 16:**
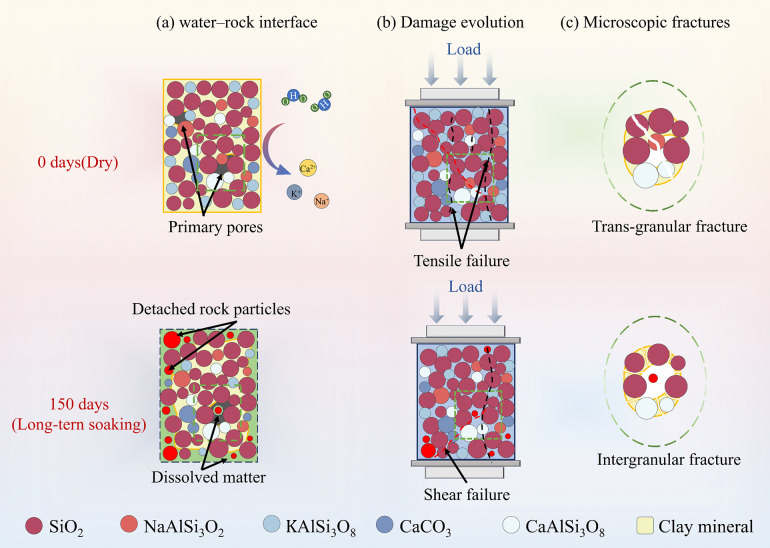
Schematic diagram of red sandstone deterioration due to water.

According to the XRD test results (Fig 3), red sandstone is mainly composed of quartz, feldspar and clay minerals. With increased soaking time, feldspar particles and clay minerals dissolve, while quartz particles do not change significantly [59]. The mineral composition of quartz shows an increasing trend. The presence of quartz particles leads to a higher probability of splitting failure [60]. Furthermore, the continuous dissolution of cementitious materials gradually weakens the bonding between mineral particles, reduces the effective load-bearing area among particles, and promotes the expansion of microcracks and voids. Additionally, ions released during feldspar dissolution alter the chemical environment of pore water, accelerating the deterioration of red sandstone’s mechanical properties (Fig 16a). SEM test results further confirm that mineral dissolution during soaking gradually blurs the contours of mineral particles. With the increase in soaking duration, debris generated from mineral particles via hydration gradually dissolves and detaches, and the space previously occupied by the debris is replaced by pores, thereby leading to a continuous increase in the porosity of red sandstone. Intergranular fractures increase with water–rock interactions, and the energy required for trans-granular fractures is greater than that for intergranular fractures [61]. This indicates that with increased soaking time, less and less energy is required, showing a negative correlation with the degree of intergranular fractures.

In summary, the combined physical and chemical effects of water are the reasons for the changes in the mechanical properties, fracture modes, and energy evolution laws of red sandstone. This result is quite similar to existing research findings [18,62,63]. As the soaking time increases, the secondary porosity of the sample also increases, the area of water rock interaction becomes larger, and the degree of binding between mineral particles decreases. The internal skeleton of the rock sample gradually becomes weak, and its brittleness decreases accordingly. The failure mode changes from brittleness to plasticity.

The damage constitutive model established in this study only incorporates the damage evolution laws under soaking and uniaxial compression, with certain limitations remaining in the research. The actual in-situ water environment and stress conditions of rocks are often more complex, whereas the uniaxial compression tests and distilled water soaking tests employed in this study are significantly simplified compared with real engineering conditions. This consequently restricts the applicability of the established model in practical engineering scenarios. Future research will be dedicated to developing a rock damage constitutive model applicable to multi-load conditions, so as to enhance the guiding value of the model for engineering practice.

## 6. Conclusions

Through multi–method experiments and theoretical modeling, this study systematically explored the energy dissipation mechanism and damage evolution law of red sandstone softening under different soaking times. A damage constitutive model considering the nonlinear characteristics of the initial compaction was established. The results provide key support for research into the multi–scale damage mechanisms of soft rock in water, as well as its engineering applications. The relevant conclusions are as follows:

(1) During soaking, the content of clay minerals in red sandstone decreases due to swelling and loss, while the proportion of quartz particles relatively increases. In terms of the meso-structure, pores and fissures continue to develop, the particle contact mode gradually changes from face–to–face to line–to–face contact, and the fracture mechanism gradually evolves, shifting from trans-granular fracture to intergranular fracture domination This lays a structural foundation for macroscopic mechanical property deterioration.
(2) With increased soaking duration, the uniaxial compressive strength and elastic modulus of red sandstone decrease stepwise. When soaked for 150 days, the cumulative deterioration degrees reach 44.25% and 30.78%, respectively, and the deterioration rate significantly accelerates when soaked for 60 days. Mechanical property deterioration is significantly positively correlated with soaking duration, verifying the time–varying effect of water–rock interactions.
(3) As the soaking time increases, the proportion of elastic strain energy in the total energy gradually decreases, and the turning point of dissipated energy moves forward. The erosive effect of water weakens the energy storage capacity of the sample. The loosening of the microstructure of rock samples leads to changes in the energy dissipation path, accelerating the damage process and forming a coupling effect of “structural degradation energy dissipation enhancement damage intensification”.
(4) By introducing the correction coefficient *K*, a damage constitutive model considering the nonlinear section of initial compaction was constructed. The nonlinear relationship between *K* and strain was described by a power function, which effectively eliminated the prediction deviation of the traditional model in the compaction stage. The theoretical curve has a high degree of fitting with the test curve, accurately characterizing the damage evolution law under different soaking durations. This model provides a quantitative tool for long–term stability assessment in soft rock engineering, promoting the development of energy–damage theory under water–rock interactions.

## Supporting information

S1 File Supplementary material. (XLSX)
